# Circulating irisin is linked to bone mineral density in geriatric Chinese men

**DOI:** 10.1515/med-2020-0215

**Published:** 2020-08-03

**Authors:** Jianmei Zhang, Xiaocheng Huang, Rongbo Yu, Yan Wang, Congcong Gao

**Affiliations:** Department of Geriatrics, Weihai Municipal Hospital Affiliated to Shandong University, 76 Heping Rd, Weihai, Shandong, 264200, P. R. China

**Keywords:** irisin; bone mineral density; osteopenia; osteoporosis

## Abstract

**Background:**

While there is evidence of a link between irisin and bone metabolism, prior clinical evaluations have been limited to women with postmenopausal osteoporosis. The primary goal of this evaluation is to examine the relationship between irisin and bone mineral density (BMD) in geriatric Chinese men experiencing osteoporosis or osteopenia.

**Methods:**

In this case–control study, 43 geriatric Chinese men were verified as having osteoporosis or osteopenia via dual-energy X-ray light absorption spectrophotometry, and 24 subjects were accepted as the controls. Serum irisin levels were detected by a commercial ELISA kit.

**Results:**

Serum irisin levels were lowered in geriatric Chinese men with osteopenia and osteoporosis, and multiple linear regression analysis revealed that the serum irisin level is an independent factor impacting BMD.

**Conclusions and discussion:**

Our data confirm a positive correlation between irisin levels and BMD in geriatric Chinese men. Irisin has a protective effect on bone health dependent on BMD, but large clinical trials are still required to verify the irisin and BMD relationship.

## 1 Introduction

Osteoporosis is an age-related systemic and progressive disease, which is caused by an increase in osteoclastic bone resorption and/or reduced osteoblastic bone formation. A fracture is the gravest complication of osteoporosis, which is the major cause of morbidity and mortality in geriatric patients [1].

The bone–muscle unit has received attention based on the very tight connection of bone mass/geometry and muscles. Some research studies [2,3] have shown that muscle mass was positively linked to bone mineral density (BMD) and lowering fracture risk; these outcomes reinforced the idea that the bone–muscle unit is the functional unit [4]. As for the impacts of muscle on bone metabolism, myokines and growth factors, which are derived from skeletal muscle cells, are thought to have pivotal roles [3,5].

Of these myokines, the recently noted myokine irisin is of the most interest. Irisin was distinguished in 2012, and it is secreted from skeletal muscle and liberated into circulation during physical exercise in mice and humans [6]. Irisin was first noted as an activator of adipose tissue browning, so it has an impact on battling obesity and diabetes [7,8,9].

Current evaluations have revealed that irisin is also tightly linked to bone metabolism. In an *in vivo* evaluation, recombinant irisin administration increased cortical bone mass and strength by inducing bone formation but led to fewer osteoclasts in male mice [10]. Colaianni et al. [11] revealed that irisin induced osteoblast differentiation, partially via the bone morphogenetic protein pathway, and prevented osteoclast differentiation by subduing the RANKL-Akt1/MITF/PU1-NFATc1 pathway. A prior evaluation in humans revealed that serum irisin levels are connected to the incidence of osteoporotic fractures in postmenopausal women with osteopenia [12]. These discoveries indicate that irisin could be a helpful marker for evaluating muscle/bone disorders and metabolic diseases.

Nevertheless, the evaluation of the relationship between serum irisin and BMD is still limited to females with postmenopausal osteoporosis. Comparable evaluations in men with osteoporosis still need to be conducted. We hypothesized that the level of circulating irisin was also negatively correlated with BMD in elderly men. In this evaluation, we discovered that the serum levels of irisin were lowered in geriatric males with osteoporosis and osteopenia. Further regression analysis showed that irisin reduction was an independent risk factor for BMD.

## 2 Patients and methods

### 2.1 Study population

**Figure 1:**
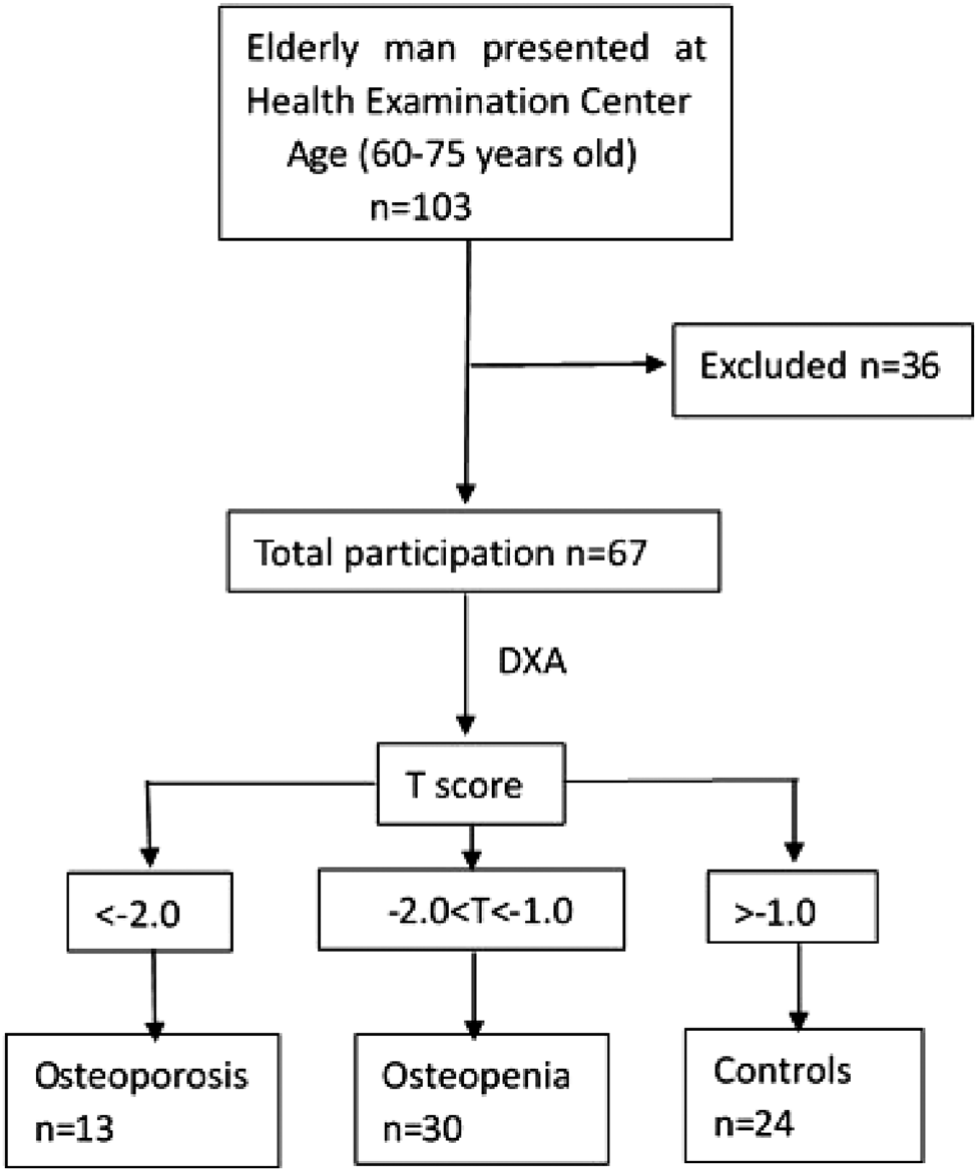
The inclusion and exclusion of the population.

Geriatric Chinese men were consecutively enrolled at the Health Examination Center of Weihai Municipal Hospital, Weihai, China. The recruitment process and research design are summarized in Figure 1.

BMD at the LS and/or the nondominant FN was tested by dual-energy X-ray light absorption spectrophotometry. Geriatric man with *T*-score ≤−2.0 served as patients with osteoporosis, those with *T*-score > −2.0 but < −1.0 as patients with osteopenia, and *T*-score > −1.0 as controls.

Exclusion criteria for all groups were as follows: (1) age < 60 years; (2) any bone and mineral disease other than primary osteoporosis, including Paget’s disease, osteogenesis imperfecta, rheumatologic diseases, primary and secondary hyperparathyroidism, paralysis, and chronic immobilization; (3) any musculoskeletal injuries or surgical history in past 2 years; (4) myopathy or systematic diseases that may affect the muscles; (5) any medications that could affect bone and muscle metabolism including corticosteroids, nonsteroidal anti-inflammatory drugs, statins, thiazolidinediones, interferon, metronidazole, tamoxifen, immunosuppressive agents, anticonvulsants, antiviral drugs, anti-tuberculosis agents, and addictive drugs; (6) severe liver or kidney disease; (7) liver or kidney transplantation; (8) any malignancy; (9) uncontrolled thyroid disease; and (10) dental surgery in past 6 months.

The minimum number of cases we need to include is according to the formula: *n* = (*U*_*α*_ + *U*_*β*_)2 × (1 + 1/*κ*) × *σ* ^2^/*δ* ^2^, where *U*_*α*_ is the *U* value of the first type of error probability (*α* = 0.05), *U*_*β*_ is the *U* value of the second type of probability error (*β* = 0.1), *δ* is the absolute value of the mean difference between the experimental group and the control group, *σ* ^2^ is the total variance, which is estimated by the sample variance *S* ^2^, *S* ^2^ = (*S* ^2^_e_ + *S* ^2^_c_)/2, *S*_e_ and *S*_c_ were the standard deviation of experimental group and control group, respectively.

This study was approved by the ethics committee of the Weihai Municipal Hospital, and informed consent was signed before blood samples were collected.

### 2.2 Methods

Baseline assessments included the following: (1) detailed medical history collection including habits and daily exercise, physical examination, BMI calculation; (2) BMD at the LS and/or the nondominant FN was tested by dual-energy X-ray light absorption spectrophotometry; (3) fasting blood of controls and patients was taken from 6 am to 7 am, and serum levels of total calcium (Ca), phosphate (P), and total alkaline phosphatase (tALP) were measured within an hour after drawing blood using standard methods; (4) these samples were sent to the Central Laboratory of Weihai Municipal Hospital, and 1,25-hydroxyvitamin D, N-terminal osteocalcin (N-MID), β Crosslaps (β-CTX), N-terminal pro-peptide of type I collagen (PINP), testosterone, and parathyroid hormone (PTH) were measured by the method of ELISA; and (4) additional samples were centrifuged immediately, and the serum was separated and stored at −80°C.

Serum irisin was detected by a commercial ELISA kit (CUSABIO, Wuhan, China); intra-assay coefficient of variation (CV) was 6.5%, inter-assay CV was 8.7%, and lower limit of quantitation was 3.12 ng/mL. The professional soft “Curve Expert” was used by the supplier to make a standard curve and the OR = 0.999. For the samples that generated values higher than the highest standard, we diluted the samples with sample diluent and repeated the assay.

### 2.3 Statistical analysis

Data for continuous variables are shown as mean ± standard deviation (SD) of the mean. The Kolmogorov–Smirnov test was utilized to confirm the normality of distributions of the continuous variables. One-way ANOVA and least significance difference post hoc test were utilized for comparisons among groups. Multiple linear regression analysis (enter method) was utilized to determine variables that were independently connected to serum irisin levels. A two-sided *p* value of less than 0.05 was considered to be statistically significant in each of the above-mentioned tests. Statistical analysis was conducted with SPSS 19.0.

## 3 Results

### 3.1 Baseline characteristics of the subjects

Sixty-seven Chinese elderly men were included in this study as detailed in the study population (Figure 1). Thirteen of the subjects were assigned to the osteoporosis group, 30 were assigned to the osteopenia group, and 24 were assigned to the control group.

There was no difference in age and BMI among three groups. As anticipated, the patient groups had lowered BMD and T-scores when compared with the controls at baseline, and in contrast to the osteopenia group, BMD and T-scores were even more decreased in the osteoporosis group (LS_BMD 0.88 ± 0.09*a* vs. 0.79 ± 0.06*b* vs. 0.67 ± 0.03, *p* < 0.001; FN_BMD 0.58 ± 0.17 vs. 0.48 ± 0.02 vs. 0.37 ± 0.04, *p* < 0.001). Despite a decreasing trend in the markers of bone metabolism including N-MID (13.80 ± 3.23 vs. 12.85 ± 3.69 vs. 11.31 ± 4.24 ng/mL, *p* = 0.148), PINP (37.12 ± 8.38 vs. 34.45 ± 9.91 vs. 30.87 ± 8.41 ng/mL, *p* = 0.142), and 25-hydroxyvitamin D (27.72 ± 13.67 vs. 24.37 ± 7.52 vs. 21.96 ± 8.90  ng/mL, *p* = 0.177) in the osteopenia and osteoporosis groups at baseline, there was no statistically significant difference between the groups. Other indicators including β-CTX, testosterone, PTH, calcium, phosphorus, and magnesium remain unaltered between the patient groups and the control group (Table 1).

**Table 1:** Baseline comparative data of osteoporosis, osteopenia, and control groups

Variable	Group	Baseline	*p*-value within groups	*p*-value between groups
Age	Control	63.96 ± 5.98^a^	0.869	0.187
	Osteopenia	66.20 ± 6.07^b^	0.444	
	Osteoporosis	67.92 ± 8.14^c^	0.351	
BMI	Control	26.69 ± 3.01^a^	0.825	0.641
	Osteopenia	26.53 ± 2.41^b^	0.995	
	Osteoporosis	25.82 ± 2.88^c^	0.774	
LS_BMD (g/cm^2^)	Control	0.88 ± 0.09^a^	< 0.001	< 0.001
	Osteopenia	0.79 ± 0.06^b^	0.026	
	Osteoporosis	0.67 ± 0.03^c^	< 0.001	
LS_T-score (SD)	Control	0.83 ± 2.90^a^	< 0.001	< 0.001
	Osteopenia	−1.83 ± 0.40^b^	0.007	
	Osteoporosis	−3.64 ± 0.73^c^	< 0.001	
FN_BMD (g/cm^2^)	Control	0.58 ± 0.17^a^	< 0.001	< 0.001
	Osteopenia	0.48 ± 0.02^b^	0.032	
	Osteoporosis	0.37 ± 0.04^c^	< 0.001	
FN_T-score (SD)	Control	0.40 ± 0.290^a^	< 0.001	< 0.001
	Osteopenia	−1.63 ± 0.40^b^	0.001	
	Osteoporosis	−3.44 ± 0.73^c^	< 0.001	
N-MID (ng/mL)	Control	13.80 ± 3.23^a^	0.605	0.148
	Osteopenia	12.85 ± 3.69^b^	0.688	
	Osteoporosis	11.31 ± 4.24^c^	0.213	
25(OH)D (ng/mL)	Control	27.72 ± 13.67^a^	0.68	0.177
	Osteopenia	24.37 ± 7.52^b^	0.205	
	Osteoporosis	21.96 ± 8.90^c^	0.766	
PINP (ng/mL)	Control	37.12 ± 8.38^a^	0.556	0.142
	Osteopenia	34.45 ± 9.91^b^	0.65	
	Osteoporosis	30.87 ± 8.41^c^	0.115	
β-CTX (ng/mL)	Control	0.33 ± 0.13^a^	0.996	0.962
	Osteopenia	0.34 ± 0.19^b^	0.991	
	Osteoporosis	0.33 ± 0.16^c^	1	
Testosterone (ng/mL)	Control	5.55 ± 2.10^a^	0.984	0.334
	Osteopenia	4.78 ± 1.88^b^	0.426	
	Osteoporosis	4.95 ± 1.51^c^	0.687	
PTH (pg/ml)	Control	39.50 ± 14.40^a^	0.763	0.425
	Osteopenia	34.59 ± 11.09^b^	0.475	
	Osteoporosis	40.76 ± 22.91^c^	0.527	
Calcium (mmol/L)	Control	2.28 ± 0.08^a^	0.994	0.504
	Osteopenia	2.26 ± 0.07^b^	0.527	
	Osteoporosis	2.26 ± 0.10^c^	0.914	
Phosphorus (mmol/L)	Control	0.97 ± 0.12a	0.763	0.324
	Osteopenia	0.93 ± 0.11b	0.367	
	Osteoporosis	0.96 ± 0.14c	0.995	
Magnesium (mmol/L)	Control	0.84 ± 0.06^a^	0.843	0.555
	Osteopenia	0.88 ± 0.28^b^	0.651	
	Osteoporosis	0.84 ± 0.05^c^	0.814	
tALP (U/L)	Control	52.70 ± 9.94^a^	1	0.98
	Osteopenia	53.22 ± 12.40^b^	0.998	
	Osteoporosis	53.38 ± 10.50^c^	0.996	

Data are presented as mean ± standard deviation (SD). BMI: body mass index; BMD: bone mineral density; N-MID: N-terminal osteocalcin; 25(OH)D: 25-hydroxyvitamin D; PINP: procollagen type 1 N-terminal pro-peptide; β-CTX: β Cross-laps; PTH: parathyroid hormone; tALP: total alkaline phosphatase.

^a^Osteopenia group compared with the controls.

^b^Osteopenia group compared with the osteoporosis group.

^c^Osteoporosis group compared with the controls.

### 3.2 Association between bone density and serum irisin levels

**Figure 2:**
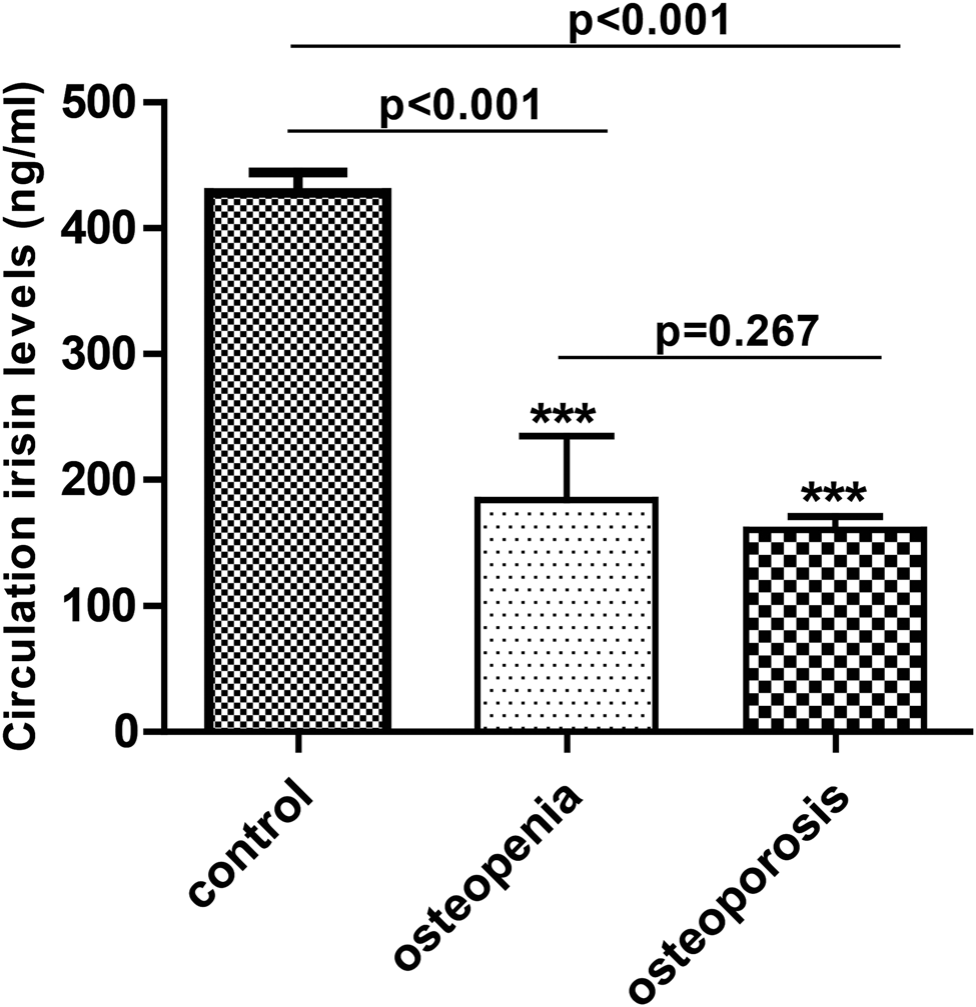
The serum irisin levels in the control, osteopenia, and osteoporosis groups.

At baseline, the serum irisin levels were significantly lowered in patients with osteoporosis and osteopenia in contrast to the controls (159.68 ± 41.08 vs. 184.37 ± 51.20 vs. 422.13 ± 95.22, *p* < 0.001), and additional within-group comparisons revealed no differences among the osteopenia and osteoporosis groups (159.68 ± 41.08 vs. 184.37 ± 51.20, *p* = 0.267) (Figure 2).

We performed multiple linear regression analysis to further investigate the association between BMD and serum irisin levels (Table 2). The variables including age, BMI, N-MID, 25(OH)D, PINP, β-CTX, PTH, testosterone, calcium, phosphorus, irisin, and tALP were all added to the multiple linear regression model with the enter stepwise method. The analysis demonstrated that calcium, N-MID, and serum irisin levels significantly contributed to the BMD (*p* < 0.001, 0.002, 0.005, respectively).

**Table 2:** Multiple linear regression analysis associated with BMD

Variables	*β*	*t*	*p*
Calcium	0.456	3.690	0.001
Irisin	0.207	3.295	0.002
N-MID	0.336	2.881	0.005

The variables calcium, irisin, and N-MID were significant (*p* < 0.05) in the multiple linear regression analysis; the other variables of interest including age, BMI, 25(OH)D, PINP, β-CTX, PTH, testosterone, phosphorus, and tALP were not significant in the model by the enter stepwise method. *β*: standardized regression coefficient.

### 3.3 Connection of circulating irisin levels and variables

We additionally analyzed the connection between irisin and other variables by multiple linear regression analysis, and our data revealed that circulating irisin levels were connected to PINP independent of age, BMI, 25(OH)D, N-MID, β-CTX, PTH, testosterone, calcium, phosphorus, and tALP (Table 3).

**Table 3:** Multiple linear regression analysis of irisin associated with other variables

Variables	*β*	*t*	*p*
Age	−1.044	−1.903	0.063
BMI	0.86	1.403	0.167
N-MID	−0.380	−1.197	0.340
25(OH)D	−0.179	−0.962	0.237
PINP	1.037	2.932	0.005
β-CTX	−0.466	−1.934	0.059
PTH	0.148	0.712	0.480
Testosterone	0.068	0.054	0.957
Calcium	0.169	0.330	0.743
Phosphorus	0.060	0.241	0.811
tALP	0.080	0.248	0.805

Variables that showed significant association with circulating irisin levels and variables of interest were all included in this multiple linear regression analysis by the stepwise method. *β*: standardized regression coefficient.

## 4 Discussion

In this evaluation, we initially documented that serum irisin levels were lowered in aged Chinese men with osteopenia and osteoporosis, and multiple linear regression analysis revealed that serum irisin levels, calcium, and N-MID are independent factors impacting BMD.

Irisin is a myokine secreted from skeletal muscle into circulation and increased significantly following physical activities [6]. Quite a lot of studies [13] have shown that physical exercise could promote bone formation and has a beneficial effect on BMD. Skeletal muscle and bone are considered as functional units [14] in recent years; since irisin was discovered, it has been hypothesized as a possible key factor by which physical exercise may exert the protective effect on bone tissue.

A small observational clinical study demonstrated that irisin levels were connected to osteoporotic fractures in patients with postmenopausal osteoporosis or osteopenia [14]. And the study of Anastasilakis et al. [12] came to the same conclusion. In female athletes, irisin levels were lowered in young amenorrheic athletes and positively connected to bone density and strength [15]. Male mice injected with r-irisin were found to have an improved cortical bone mass and geometry [11]. In an *in vitro* evaluation, osteoblasts differentiated in the presence of conditioned medium from exercised muscle expressed a higher level of ALP and collagen type I mRNA, but the impact was fully obstructed when a neutralizing antibody against irisin was put into the conditioned medium [10]. These discoveries showed that bone tissue is one of the target organs of irisin, and it has a protective role in bone health.

Colaianni et al. [11] performed a systematic study of the effect and its potential mechanism of irisin on bone tissues. Their data showed that irisin can increase bone formation parameters, such as bone formation rate and mineral apposition rate, by inducing osteoblast activity. The possible mechanism was involved in phosphorylated Erk, then it upregulated Atf4, Runx2, Osx, Lrp5, β-catenin, Alp, and Col1a1.

The outcomes revealed that circulating irisin levels were lowered significantly in elderly Chinese males with osteopenia or osteoporosis, and multiple regression analysis showed that irisin levels were connected to BMD.

Our results support the current opinion that irisin is a protective factor of bone tissue.

In addition, our data exhibited that irisin levels were independent of advancing age, BMI, 25(OH)D, and PTH levels, which was consistent with previous reports [7,12,16].

Our study has the following limitations: (1) the sample size was small, (2) it was not a double-blinded and randomized trial, and (3) we did not establish lean body mass or BMD in the patients.

In conclusion, our data showed that circulating irisin levels were significantly decreased in geriatric Chinese males with osteopenia or osteoporosis, and irisin levels were connected to BMD. Additional evaluations are required to determine if irisin can function as an independent predictor for osteoporosis and osteoporotic fracture.
